# A comparison of quality of abstracts of systematic reviews including meta-analysis of randomized controlled trials in high-impact general medicine journals before and after the publication of PRISMA extension for abstracts: a systematic review and meta-analysis

**DOI:** 10.1186/s13643-016-0356-8

**Published:** 2016-10-13

**Authors:** Jean Joel R. Bigna, Lewis N. Um, Jobert Richie N. Nansseu

**Affiliations:** 1 Department of Epidemiology and Public Health, Centre Pasteur of Cameroon, Yaoundé, Cameroon; 2 Faculty of Medicine and Biomedical Sciences, University of Yaoundé I, Yaoundé, Cameroon; 3 Sickle Cell Disease Unit, Mother and Child Centre of the Chantal Biya Foundation, Yaoundé, Cameroon

**Keywords:** Abstract, PRISMA, Systematic review, Randomized controlled trial, Meta-analysis, General medicine journal

## Abstract

**Background:**

Journal abstracts including those reporting systematic reviews (SR) should contain sufficiently clear and accurate information for adequate comprehension and interpretation. The aim was to compare the quality of reporting of abstracts of SRs including meta-analysis published in high-impact general medicine journals before and after publication of the Preferred Reporting Items for Systematic Reviews and Meta-Analyses (PRISMA) extension for abstracts (PRISMA-A) released in April 2013.

**Methods:**

SRs including meta-analysis of randomized controlled trials published in 2012, 2014, and 2015 in top-tier general medicine journals were searched in PubMed. Data was selected and extracted by two reviewers based on the PRISMA-A guidelines which recommend to include 12 items. The primary outcome was the adjusted mean number of items reported; the secondary outcome was the reporting of each item and factors associated with a better reporting. Adjustment was made for abstract word count and format, number of authors, PRISMA endorsement, and publication on behalf of a group.

**Results:**

We included 84 abstracts from 2012, 59 from 2014, and 61 from 2015. The mean number of items reported in 2015 (7.5; standard deviation [SD] 1.6) and in 2014 (6.8; SD 1.6) differed and did not differ from that reported in 2012 (7.2; SD 1.7), respectively; adjusted mean difference: 0.9 (95 % CI 0.4; 1.3) and −0.1 (95 % CI −0.6; 0.4). From 2012 to 2014, the quality of reporting was in regression for “strengths and limitations of evidence” and “funding”; contrariwise, it remained unchanged for the others items. Between 2012 and 2015, the quality of reporting rose up for “description of the effect”, “synthesis of results”, “interpretation”, and “registration”; but decreased for “strengths and limitations of evidence”; it remained unchanged for the other items. The overall better reporting was associated with abstracts structured in the 8-headings format in 2014 and abstracts with a word count <300 in 2014 and 2015.

**Conclusions:**

Not surprisingly, the quality of reporting did not improve in 2014 and suboptimally improved in 2015. There is still room for improvement to meet the standards of PRISMA-A guidelines. Stricter adherence to these guidelines by authors, reviewers, and journal editors is highly warranted and will surely contribute to a better reporting.

**Electronic supplementary material:**

The online version of this article (doi:10.1186/s13643-016-0356-8) contains supplementary material, which is available to authorized users.

## Background

Systematic reviews and meta-analyses of randomized controlled trials (RCTs) are fundamental tools which can be used to generate reliable summaries of health care information directed to clinicians, decision makers, and patients as well [1]. RCTs by their design generally provide the best quality of evidence required for health care decisions about interventions compared to observational studies [2, 3]. As such, they should be reported according to the highest possible pre-defined standards, as well as their systematic reviews and meta-analyses. Indeed, systematic reviews of RCTs provide information on clinical benefits and harms of interventions, inform the development of clinical recommendations, and help to identify future research needs. Besides, they are the reference standard for synthesizing evidence in healthcare about interventions because of their methodological rigor [4]. Clinicians read them to keep up to date with their field [5, 6]. As with any other research, the value of a systematic review depends on what was done, what was found, and the clarity of its reporting. Like other publications, low quality of reporting of systematic reviews can limit readers’ ability to assess the strengths and weaknesses of these reviews.

With an overwhelming day-to-day workload, the availability of large volumes of scientific publications, limited access to many full-text articles, healthcare professionals often make recourse to information contained in abstracts to guide or sustain their decisions [7, 8]. Within queries to PubMed, most readers look only at titles; only half of searches result in any click on content [9]. What makes matters worse, the average number of titles clicked on to obtain the abstract or full text, even after retrieving several searches in a row, is less than five. Of those clicks, abstracts will be represented about 2.5 times more often than full texts [9].

Abstracts can be useful for: screening by study type, facilitating quick assessment of validity, enabling efficient perusal of electronic search results, clarifying to which patients and settings the results apply, providing readers and peer reviewers with explicit summaries of results, facilitating the pre-publication peer review process, and increasing precision of computerized searches [10–13]. Consequently, journal and conference abstracts must contain sufficiently clear and accurate information that will permit adequate comprehension and interpretation of systematic reviews and meta-analyses findings [14].

After observing that the quality of abstracts of systematic reviews is still poor [15], the Preferred Reporting Items for Systematic Reviews and Meta-analyses (PRISMA) for Abstracts Group developed an extension to the PRISMA Statement in order to provide guidance on how to write and present abstracts for systematic reviews and meta-analyses [14]. These guidelines were published in April 9 2013. To the best of our knowledge, there is no published study that has scrutinized the impact of release of PRISMA for abstracts (PRISMA-A) yet, comparing the quality of abstracts of papers published before (2012) and after (2014 and 2015) this release. Consequently, in an effort to promote quality reports of abstracts in systematic reviews and meta-analyses of RCTs, we firstly compared the quality of reporting abstracts in high-impact biomedical journals, before and after publication of PRISMA guidelines for abstracts. The secondary objectives included comparing the quality of reporting of each item of PRISMA-A guidelines before and after the publication of PRISMA-A. We also aimed to investigate factors associated with better adherence to PRISMA-A guidelines.

## Methods

### Design

We identified abstracts of systematic reviews published in 2012, 2014, and 2015 in high-impact general medicine journals. We assessed the reporting of these abstracts according to PRISMA-A recommendations [14]. We compared reporting from 2012 (before PRISMA-A) with reporting in 2014 and 2015 (after PRISMA-A). The present review was not registered. The PRISMA guidelines served as the template for reporting the present review [16]. The PRISMA checklist can be found in the Additional file 1.

### Data sources

Based on impact factor published in 2015 by Thomson Reuters [17], the top nine high-impact general medical journals were selected for this study, namely: New England Journal of Medicine (NEJM), The Lancet, Journal of American Medical Association (JAMA), Annals of Internal Medicine, British Medical Journal (BMJ), Archives of Internal Medicine, PLOS Medicine, JAMA Internal Medicine, BMC Medicine, and Mayo Clinic Proceedings. In the PRISMA website, NEJM, Annals of Internal Medicine, Archives of Internal Medicine, and Mayo Clinic Proceedings are not cited as endorsers of PRISMA guidelines by contrast to the other journals which are PRISMA endorsers [18]. Journals were not excluded on the basis of their lack of endorsement of the PRISMA statement. We conducted a PubMed search of all systematic reviews and meta-analyses published in years 2012, 2014, and 2015. Our search strategy included “meta-analysis” as publication type, journal names, and limits were set for the specific time periods of interest (2012/01/01 to 2012/12/31, 2014/01/01 to 2014/12/31, and 2015/01/01 to 2015/12/31). The search strategy is presented in the Additional file 2.

### Studies selection and data extraction

Two reviewers (JJRB and LNU) independently selected abstracts of systematic reviews of RCTs including meta-analyses. A “yes” or “no” answer was assigned to each item indicating whether the authors had reported it or not. Data extraction was independently performed by two reviewers (JJRB and LNU) in compliance with recommendations of the evaluation of PRISMA-A guidelines [14]. This was done for the 12 items recommended by the PRISMA-A guidelines. Agreement was measured using the Kappa statistic [19]. Disagreements were resolved by consensus after discussion between authors.

In addition to the PRISMA-A items, we collected the journal name, number of authors, type of abstract format (Introduction, Methods, Results, and Discussion [IMRAD]; 8-headings [objective, design, setting, participants, intervention, main outcome measured, results, and conclusions]), PRISMA endorser journal (yes or no), and actual observed abstract word count (<300 versus ≥300).

### Outcomes measured

The primary outcome was the number of items reported among the 12 recommended items of the PRISMA-A guidelines; the secondary one was the proportion of abstracts reporting each of these 12 recommended items.

### Statistical analysis

Data were entered and analyzed using the Statistical Package for Social Science (SPSS) version 21.0 for Windows (IBM Corp. Released 2012. IBM SPSS Statistics for Windows, Version 21.0. Armonk, NY: IBM Corp.). Categorical variables were expressed as numbers (*N*) with percentages (%). Continuous variables were expressed as means with standard deviation (SD).

We expressed the number of items for each year as mean (SD) and estimated the unadjusted and adjusted differences using the independent two-sample Student *t* test and generalized estimation equations (GEE), respectively [20]. The mean differences and adjusted means were reported with their 95 % confidence intervals (95 % CI) and *p* values. For continuous variables (number of items reported, for example), we assumed a linear distribution. We compared compliance with the 12 items of the PRISMA-A for years 2012 versus 2014 and 2015 using individual chi-squared test or Fischer’s exact test where necessary. Unadjusted analysis was followed by an adjusted analysis using GEE. For binary outcomes, we assumed a binomial distribution and an unstructured correlation matrix. We reported measures of association with odds ratios (OR) for univariable analyses and adjusted odds ratios (aOR) for multivariable analyses together with their 95 % confidence intervals (CI) and *p* values. Factors associated with overall better reporting were investigated using GEE and assuming Poisson distribution for number of items reported. The magnitude of association between better reporting and investigated factors was measured with adjusted incidence rate ratio (aIRR) alongside its 95 % CI. Additionally, we conducted sensitive analyses to determine factors associated with a better quality of reporting of the “Methods” and “Results” sections of abstracts for years 2014 and 2015.

For GEE, adjustments were made for actual observed abstract word count (<300 versus ≥300), PRISMA endorser journal (yes versus no), abstract format (IMRAD versus 8-headings), publication on behalf of a group (yes versus no), number of authors (≤6 versus >6), with journal as a grouping factor to adjust for potential clustering or similarity in articles published in the same journal. Evidence against the null hypothesis was considered for a two-tailed *p* value of <0.05. The 300-word cut-off was chosen as it has been shown, like for other abstract reporting guidelines such as the Consolidated Standards of Reporting Trials (CONSORT) extension for abstracts, that a word count around 300 is sufficient to fill all items [20]. The adjustment was done for abstract format because there are previous studies reporting relationship between abstract format and quality reporting [21, 22]. The adjustment was also done for number of authors and publication on behalf a group because it was reported an association between higher quality of work and increased number of collaborators [23, 24], though some other studies did not end up with the same finding [25, 26].

## Results

### General characteristics of abstracts selected

**Fig. 1:**
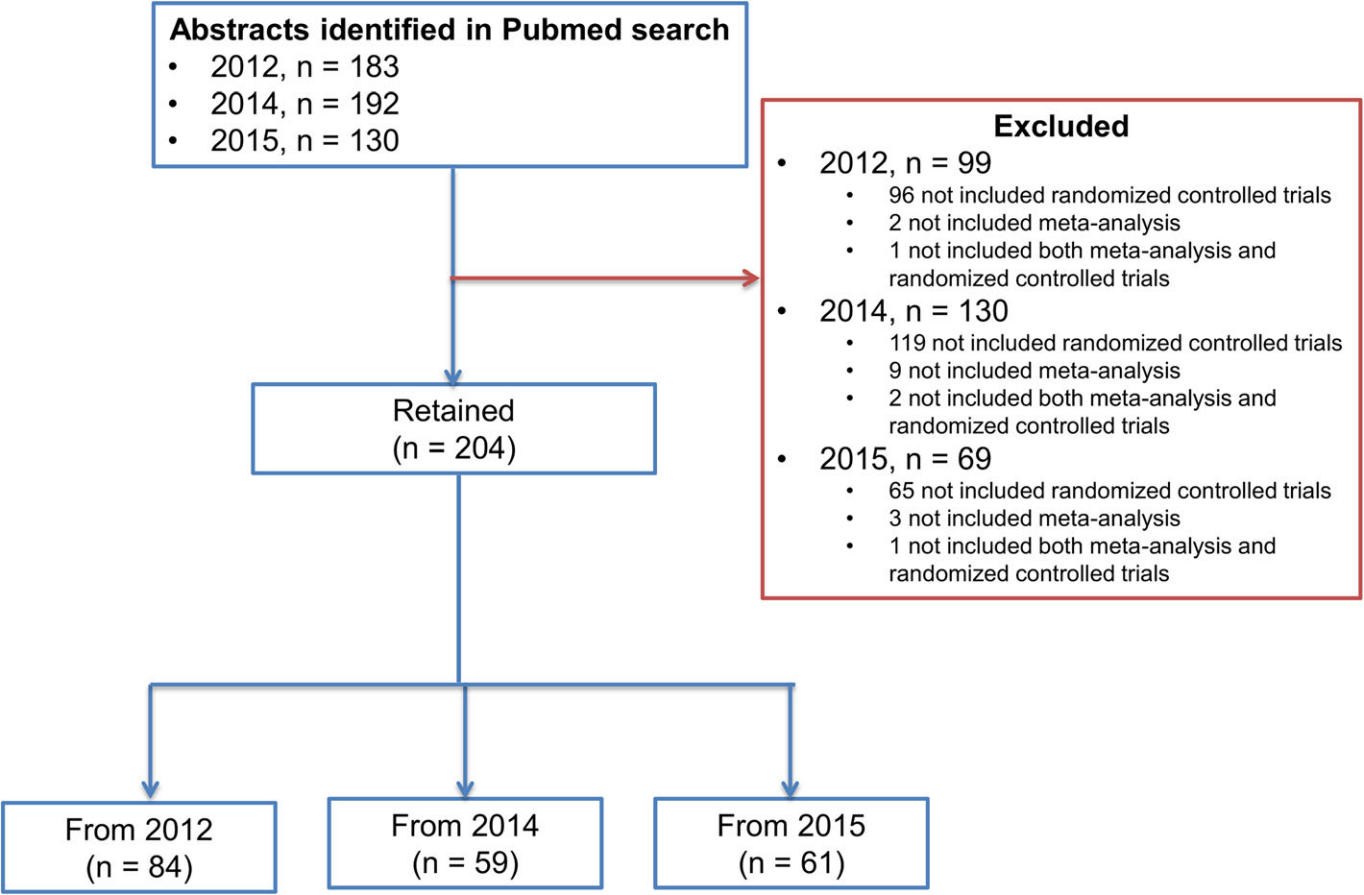
Flow chart of studies considered for inclusion

Our search yielded 505 articles of which 183 were published in 2012, 192 in 2014, and 130 in 2015. Three hundred and one abstracts did not meet our inclusion criteria, hence were excluded because most of them not included RCTs in the meta-analysis (*n* = 280). On the whole, we included 204 abstracts: 84 from 2012, 59 from 2014, and 61 from 2015 as shown in the flow diagram (Fig. 1). Agreement between reviewers on all PRISMA-A items was high (Kappa = 0.79, *p* < 0.001). Additional file 3 lists all abstracts included in this review.

As concerns the journals in which the abstracts were published, 67 (32.8 %) were published in BMJ (Table 1). The 8-headings format was used in 132 (64.7 %) abstracts. Among all the journals, 163 (79.9) were published with an actual observed abstract word count ≥300, 19 (9.3 %) abstracts reported publications on behalf of a group in authorship, and 150 abstracts (73.5 %) were from journals which endorsed PRISMA. Four of the 10 journals were PRISMA endorsers. The mean number of authors per article was 8.5 (SD 5.4) (Table 1).

**Table 1:** Distribution of abstracts by year and characteristics

	2012 *n* = 84	2014 *n* = 59	2015 *n* = 61	All *N* = 204
Journals				
- Annals of Internal Medicine	12 (14.3)	13 (22.0)	11 (18.0)	36 (17.6)
- BMC Medicine	4 (4.8)	8 (13.6)	10 (16.4)	22 (10.8)
- BMJ	31 (36.9)	18 (30.5)	18 (29.5)	67 (32.8)
- JAMA	6 (7.1)	6 (10.2)	4 (6.6)	16 (7.8)
- JAMA Internal Medicine/Archives of Internal Medicine	13 (15.5)	11 (18.6)	4 (6.6)	28 (13.7)
- Mayo Clinic Proceedings	1 (1.2)	3 (5.1)	0	4 (2.0)
- NEJM	1 (1.2)	0	0	1 (0.5)
- PLOS Medicine	6 (7.1)	0	0	6 (2.9)
- The Lancet	10 (11.9)	0	14 (23.0)	24 (11.8)
Mean number of authors	8.2 (5.8)	8.4 (5.4)	9.1 (4.9)	8.5 (5.4)
Abstract format				
- IMRAD	35 (41.7)	12 (20.3)	25 (41.0)	72 (35.3)
- 8-headings	49 (58.3)	47 (79.7)	36 (59.0)	132 (64.7)
PRISMA endorser journals				
- Yes	57 (67.9)	43 (72.9)	50 (82.0)	150 (73.5)
- No	27 (32.1)	16 (27.1)	11 (18.0)	54 (26.5)
Publication on behalf of a group				
- Yes	8 (9.5)	4 (6.8)	7 (11.5)	19 (9.3)
- No	76 (90.5)	55 (93.2)	54 (88.5)	185 (90.7)
Actual observed abstract word count				
- <300	14 (16.7)	16 (27.1)	11 (18.0)	41 (20.1)
- ≥300	70 (83.3)	43 (72.9)	50 (82.0)	163 (79.9)

Data are *n* (%) or mean (standard deviation)

### Comparison of quality of abstract reporting between 2014 and 2012 and between 2015 and 2012

The mean number of items reported in 2014 (mean = 6.8; SD = 1.6) did not statistically differ from that reported in 2012 (mean = 6.7; SD = 1.6), mean difference (MD): 0.1; 95 % CI −0.4; 0.7; *p* = 0.694. There was no statistically significant difference after adjusting for covariates among which actual observed abstract word count, PRISMA endorsement, abstract format, publication on behalf of a group, and number of authors (MD −0.1, 95 % CI −0.6; 0.4; *p* = 1.0). The mean number of items reported in 2015 (mean = 7.5; SD = 1.6) differed significantly from that reported in 2012: mean difference (MD): 0.8; 95 % CI 0.2; 1.3; *p* = 0.007. This difference remained statistically significant after adjustment for covariates (MD 0.9, 95 % CI 0.4; 1.3; *p* = 0.002) (Table 2). An exploratory comparison between abstracts released in 2015 and those released in 2014 with regard to the overall quality of reporting showed a statistically significant difference (MD 0.63, 95 % CI 0.05; 1.21; *p* = 0.034).

**Table 2:** Comparison of mean of PRISMA for abstracts items reported in abstracts of meta-analyses of RCT

Variables	*N*	Univariable analysis^a^			Multivariable analysis^b^		
		Means (standard deviation)	Mean difference (95 % CI)	*p* value	Adjusted means (95 % CI)	Adjusted mean difference (95 % CI)	*p* value
Year							
- 2012 (ref)	84	6.7 (1.6)			7.0 (6.5; 7.5)		<0.001^*^
- 2014	59	6.8 (1.6)	0.1 (−0.4; 0.7)	0.694	6.9 (6.4; 7.4)	−0.1 (−0.6; 0.4)	0.711^**^
- 2015	61	7.5 (1.6)	0.8 (0.2; 1.3)	0.007	7.8 (7.4; 8.3)	0.9 (0.4; 1.3)	0.001^***^
Abstract word count							
- <300 (ref)	30	8.3 (2.0)			6.9 (6.3; 7.4)		
- ≥300	113	6.7 (1.4)	−0.3 (−1.3; −1.0)	<0.001	7.6 (6.8; 8.4)	0.7 (−0.4; 1.8)	0.184
PRISMA endorsement							
- Non endorser journals (ref)	43	7.9 (1.9)			7.7 (7.1; 8.2)		
- Endorser journals	100	6.6 (1.4)	−1.3 (−1.9; −0.7)	<0.001	6.8 (6.2; 7.4)	−0.8 (−1.7; 0.04)	0.063
Abstract format							
- IMRAD (ref)	47	6.4 (1.6)			6.9 (6.3; 7.4)		
- 8-headings	96	7.3 (1.6)	0.8 (0.4; 1.3)	<0.001	7.6 (7.2; 8.0)	0.7 (0.2; 1.2)	0.008
Publication on behalf of a group							
- No (ref)	131	7.0 (1.7)			7.3 (7.0; 7.7)		
- Yes	12	6.7 (1.4)	−0.3 (−1.1; 0.4)	0.394	7.1 (6.5; 7.8)	−0.2 (−0.8; 0.5)	0.557
Number of authors							
- ≤6 (ref)	64	6.9 (1.6)	0.1 (−0.4; 0.6)	0.659	7.2 (6.7; 7.6)		
- >6	79	7.0 (1.7)			7.3 (6.9; 7.7)	0.2 (−0.3; 0.6)	0.464

*Ref* reference for mean difference calculation, *PRISMA* preferred reporting items for systematic review and meta-analysis, *IMRAD* introduction, methods, results, and discussion

^a^Student’s *t* test, ^*^Global *p* value

^b^Generalized estimation equations with journals as grouping variable. Goodness of Fit: value of the Corrected Quasi-likelihood under Independence Model Criterion = 1827.12 and the value of the Quasi-likelihood under Independence Model Criterion = 1829.28

After Bonferroni correction for multiple comparisons, values are ^**^1.0 and ^***^ 0.002

Seven items were reported in more than 50 % of the abstracts: title, objectives, eligibility criteria, included studies, synthesis of results, description of effect, and interpretation. Four items were reported in less than 50 % of the abstracts: information sources, strengths and limitations of evidence, funding, and registration. Reporting of risk of bias varied, around 50 % throughout years (49.2–67.8 %) (Table 3).

**Table 3:** Reporting quality of items of the PRISMA for abstracts of meta-analyses of RCT

Items	Criteria	2015 *n* = 61	2014 *n* = 59	2012 *n* = 84
Title	Identify the report as a meta-analysis	60 (98.4)	58 (98.3)	78 (92.9)
Objectives	The research question including components such as participants, interventions, comparators, and outcomes	52 (85.2)	45 (76.3)	68 (81.0)
Eligibility criteria	Study and report characteristics used as criteria for inclusion	53 (86.9)	51 (86.4)	76 (90.5)
Information sources	Key databases searched and search dates	7 (11.5)	15 (25.4)	11 (13.3)
Risk of bias	Methods of assessing risk of bias	30 (49.2)	40 (67.8)	43 (51.2)
Included studies	Number and type of included studies and participants and relevant characteristics of studies	36 (59.0)	35 (59.3)	54 (64.3)
Synthesis of results	Results for main outcomes (benefits and harms), including summary measures and confidence intervals	61 (100)	42 (71.2)	57 (67.9)
Description of the effect	Direction of the effect and size of the effect in terms meaningful to clinicians and patients	46 (75.4)	34 (57.6)	51 (60.7)
Strengths and Limitations of evidence	Brief summary of strengths and limitations of evidence	12 (19.7)	18 (30.5)	33 (39.3)
Interpretation	General interpretation of the results and important implications	61 (100)	51 (86.4)	69 (82.1)
Funding	Primary source of funding for the review	24 (39.3)	12 (20.3)	24 (28.6)
Registration	Registration number and registry name	14 (23.0)	3 (5.1)	2 (2.4)

In univariable analysis, there was a statistically significant improvement in abstract reporting in 2014 compared to 2012 only for “risk of bias” (crude OR [cOR] = 2.0; 95 % CI 1.003; 4.0). After adjustment, “risk of bias” became insignificant while reporting of “strengths and limitations of evidence” (adjusted OR [aOR] = 0.23; 95 % CI 0.07–0.71) and “Funding” (aOR = 0.25; 95 % CI 0.07–0.81) became statistically associated with a lower quality in 2014 compared to 2012 (Table 4).

**Table 4:** Comparison of reporting quality of items of the PRISMA for abstracts of meta-analyses of RCT

Items	Univariable analysis^a^				Multivariable analysis^b^			
	2014 versus 2012		2015 versus 2012		2014 versus 2012		2015 versus 2012	
	Crude odds ratio (95 % CI)	*p*	Crude odds ratio (95 % CI)	*p*	Adjusted odds ratio (95 % CI)	*p*	Adjusted odds ratio (95 % CI)	*p*
Title	4.5 (0.52; 38.1)	0.240	4.6 (0.54; 39.4)	0.239	4.4 (0.55; 35.1)	0.163	4.5 (0.46; 43.7)	0.199
Objectives	0.76 (0.34; 1.7)	0.499	1.4 (0.56; 3.3)	0.499	0.78 (0.33; 1.8)	0.564	1.6 (0.66; 4.1)	0.286
Eligibility criteria	0.67 (0.24; 1.9)	0.451	0.70 (0.25; 2.0)	0.496	0.58 (0.17; 2.0)	0.390	0.75 (0.25; 2.3)	0.617
Information sources	2.3 (0.95; 5.4)	0.060	0.86 (0.31; 2.4)	0.770	2.0 (0.70; 5.5)	0.197	0.82 (0.26; 2.6)	0.740
Risk of bias	2.0 (1.003; 4.0)	0.048	0.92 (0.48; 1.8)	0.811	1.2 (0.55; 2.7)	0.625	0.79 (0.36; 1.7)	0.550
Included studies	0.81 (0.41; 1.6)	0.547	0.80 (0.41; 1.6)	0.519	1.1 (0.52; 2.2)	0.834	0.98 (0.50; 2.7)	0.956
Synthesis of results	1.2 (0.57; 2.4)	0.671	Not estimable	<0.001	1.1 (0.52; 2.5)	0.738	Not estimable	
Description of the effect	0.88 (0.45; 1.7)	0.711	2.0 (0.96; 4.1)	0.063	0.93 (0.45; 1.9)	0.849	2.7 (1.2; 6.1)	0.014
Strengths and Limitations of evidence	0.68 (0.34; 1.4)	0.281	0.38 (0.18; 0.82)	0.012	0.23 (0.07; 0.71)	0.011	0.13 (0.03; 0.66)	0.013
Interpretation	1.4 (0.07; 2.8)	0.474	Not estimable	<0.001	1.9 (0.58; 5.9)	0.297	Not estimable	
Funding	0.64 (0.29; 1.4)	0.264	1.6 (0.81; 3.3)	0.174	0.25 (0.07; 0.81)	0.021	1.5 (0.56; 4.0)	0.425
Registration	2.2 (0.36; 13.6)	0.404	12.2 (2.7; 56.1)	<0.001	1.9 (0.30; 12.4)	0.485	10.8 (2.3; 49.6)	0.002

*CI* confidence interval

^a^Chi-squared test

^b^Generalized estimation equations with journal as grouping variable: adjustment has been made for abstract word count (<300 versus ≥300), PRISMA endorser journal (yes versus no), abstract format (IMRAD versus 8-headings), publication on behalf a group (yes versus no), number of authors (≤6 versus >6)

In univariable analysis, there was a statistically significant improvement in abstract reporting in 2015 compared to 2012 for “synthesis of results” (cOR not estimable, *p* < 0.001), for “interpretation”

(cOR not estimable, *p* < 0.001), and for “registration” (cOR = 12.2; 95 % CI 2.7–56.1). The quality of abstract decreased from 2012 to 2015 concerning “strength and limitations of evidence” (cOR = 0.38; 95 % CI 0.18–0.82). After adjustment, “synthesis and results” (aOR not estimable, *p* < 0.001), “description of effect” (aOR = 2.7; 95 % CI 1.2–6.1), “interpretation” (aOR not estimable, *p* < 0.001), and “registration” (aOR = 10.8; 95 % CI 2.3–49.6) were statistically associated with a better quality of reporting in 2015 compared to 2012. “Strengths and limitations of evidence” was statistically associated with lower reporting quality in 2015 compared to 2012 (Table 4).

In 2014, factors statistically associated with overall better reporting were structuring abstracts in the 8-headings format compared to the IMRAD format (aIRR 1.26; 95 % CI 1.02–1.56) and abstract word count <300 (aIRR 1.20; 95 % CI 1.09–1.35). The sole factor statistically associated with a better reporting of the “Methods” section was abstract word count <300 (aIRR 1.43; 95 % CI 1.16–1.72). None of the researched factors was statistically associated with a better quality of reporting of the “Results” section (Table 5).

**Table 5:** Factors associated with a better reporting of items of PRISMA for abstracts published in 2014

	Overall reporting quality		Methods reporting quality		Results reporting quality	
	Adjusted incidence rate ratios (95 % confidence interval)^a^	*p*	Adjusted incidence rate ratios (95 % confidence interval)^a^	*p*	Adjusted incidence rate ratios (95 % confidence interval)^a^	*p*
Abstract word count						
<300 (ref)	1		1			
≥300	0.83 (0.74; 0.92)	<0.001	0.70 (0.58; 0.86)	<0.001	1.02 (0.78; 1.33)	0.878
Abstract format						
IMRAD (ref)	1					
8-headings	1.26 (1.02; 1.56)	0.036	1.40 (0.94; 2.10)	0.099	1.41 (0.91; 2.20)	0.126
Publication on behalf of a group						
No (ref)						
Yes	1.01 (0.88; 1.15)	0.911	0.85 (0.52; 1.38)	0.497	0.99 (0.62; 1.58)	0.965
Number of authors						
≤6 (ref)	1					
>6	1.02 (0.91; 1.14)	0.761	0.89 (0.73; 1.08)	0.242	1.05 (0.78; 1.41)	0.743

*Ref* reference for mean difference calculation, *PRISMA* preferred reporting items for systematic review and meta-analysis, *IMRAD* introduction, methods, results, and discussion

^a^Generalized estimation equations with journal as grouping variable. PRISMA endorser variable was excluded from model because it was redundant

In 2015, the sole factor statistically associated with an overall better reporting was abstract word count <300 (aIRR 1.20; 95 % CI 1.09–1.35). Factors statistically associated with a better reporting of the “Methods” section was abstract word count <300 (aIRR 1.43; 95 % CI 1.16–1.72), structuring abstract in the 8-headings format compared to the IMRAD format (aIRR 1.33; 95 % CI 1.08–1.65), and not publishing on behalf of a group (aIRR 1.37; 95 % CI 1.04–1.82). No factor was statistically associated with a better reporting of the “Results” section (Table 6).

**Table 6:** Factors associated with a better reporting of items of PRISMA for abstracts published in 2015

	Overall reporting quality		Methodological reporting quality		Results reporting quality	
	Adjusted incidence rate ratios (95 % confidence interval)^a^	*p*	Adjusted incidence rate ratios (95 % confidence interval)^a^	*p*	Adjusted incidence rate ratios (95 % confidence interval)^a^	*p*
Abstract word count						
<300 (ref)	1					
≥300	0.80 (0.74; 0.87)	<0.001	0.86 (0.59; 0.79)	<0.001	1.03 (0.87; 1.22)	0.731
Abstract format						
IMRAD (ref)	1					
8-headings	1.06 (0.96; 1.17)	0.279	1.33 (1.08; 1.65)	.008	1.04 (0.87; 1.24)	0.701
Publication on behalf of a group						
No (ref)						
Yes	0.90 (0.80; 1.00)	0.055	0.73 (0.55; 0.96)	.027	0.85 (0.64; 1.13)	0.271
Number of authors						
≤6 (ref)	1					
>6	1.04 (0.97; 1.12)	0.251	1.03 (0.89; 1.19)	.721	1.02 (0.88; 1.18)	0.790

*Ref* reference for mean difference calculation, *PRISMA* preferred reporting items for systematic review and meta-analysis, *IMRAD* introduction, methods, results, and discussion

^a^Generalized estimation equations with journal as grouping variable. PRISMA endorser variable was excluded from model because it was redundant

## Discussion

This study aimed to assess, according to the PRISMA-A checklist, differences in the quality of reporting of abstracts of systematic reviews and meta-analyses of RCTs published in top-tier general medicine journals before (in 2012) and after (in 2014 and 2015) publication of the PRISMA extension for abstracts [14]. Our findings demonstrate that the overall reporting quality of PRISMA abstracts has not improved in the 2014 era as compared to the pre-PRISMA-A era; however, there is a small improvement in 2015. There was no improvement of each of the 12 items in 2014; in fact, two items regressed. There was one regression and four improvements in 2015. The sole factor associated with better reporting was presenting abstracts following the 8-headings format.

These results are consistent with previous studies that reported inconsistencies and patterns of suboptimal reporting quality of abstracts across journals and fields of medicine over time [21, 27–35]. As demonstrated by Hopewell and colleagues, there are serious deficiencies in the reporting of abstracts of systematic reviews which make it difficult for readers to reliably assess study findings [36], although it has been bolstered that the endorsement of PRISMA guidelines increases adherence to recommendations [37].

Reporting of abstracts with the 8-headings format was associated with an overall and “Results” section better adherence to PRISMA-A guidelines in 2014. Actually, the use of structured abstracts is warranted to improve their quality of reporting [21, 22]. Surprisingly, abstract word count <300 was associated with better reporting overall, and better “Methods” section and “Results” section reporting in 2014 and 2015. This indicates that a word count of 300 is not too short to provide useful, complete, and comprehensive information. In addition, it has been demonstrated for the CONSORT guidelines that checklist items can be easily incorporated within a word count limit of 250 to 300 words [20]. We found that endorsement of PRISMA was not statistically associated with an improvement in the quality of abstracts. Therefore, editors, reviewers and authors should ensure that not only the guidelines for the reporting of full texts are respected, but also those for abstracts, as Hopewell and colleagues have figured out huge discrepancies in the quality of reporting between abstracts and full texts [36].

Our study presents some flaws. First, our study was based on a limited number of published studies. Nonetheless, it included all meta-analyses of RCT abstracts from high-impact medical journals found in PubMed. Second, our study may not be a representative sample of all medical journals because we selected only general medicine journals with high-impact factors. There is need for further studies to investigate all types of systematic reviews and screen randomly selected abstracts, not only from top-tier journals. However, as our findings show that the quality of reporting of systematic reviews including meta-analyses abstracts is suboptimal in these top journals, we can extrapolate that the quality of reporting may be lower in other journals. Hence, this call for improvement in the quality of reporting standards could perhaps be generalized.

## Conclusions

The reporting quality of abstracts of systematic reviews including meta-analyses of RCTs in leading general medicine journals did not improve in 2014 after the publication of the PRISMA-A guidelines and only improved slightly in 2015. There is still room for improvement to meet the standards of the PRISMA-A guidelines. Better structuring of abstracts and stricter adherence to the PRISMA-A by authors, reviewers, and journal editors is highly warranted.

## Additional files

Additional file 1: PRISMA reporting checklist. (DOC 63 kb)
Additional file 2: Search strategy for systematic reviews including meta-analysis of randomized controlled trials published in 2012, 2014 and 2015 in high-impact general medicine journals. (DOCX 15 kb)
Additional file 3: PMID list of included studies accompanied with journal name and year of publication. (XLSX 14 kb)

## Abbreviations

CONSORT: Consolidated Standards of Reporting Trials
GEE: Generalized estimation equations
IMRAD: Introduction, Methods, Results, and Discussion
PRISMA-A: Preferred Reporting Items for Systematic Review and Meta-Analysis for Abstracts
RCT: Randomized controlled trial
