# Supplementary material for: A comparison of quality of abstracts of systematic reviews including meta-analysis of randomized controlled trials in high-impact general medicine journals before and after the publication of PRISMA extension for abstracts: a systematic review and meta-analysis
Source: Syst Rev. 2016 Oct 13;5:174. doi: 10.1186/s13643-016-0356-8 (PMC5064935; doi:10.1186/s13643-016-0356-8)
Supplement: Additional file 2: — Search strategy for systematic reviews including meta-analysis of randomized controlled trials published in 2012, 2014 and 2015 in high-impact general medicine journals. (DOCX 15 kb) [file 13643_2016_356_MOESM2_ESM.docx]

**Additional file 2: Search strategy for systematic reviews including meta-analysis of randomized controlled trials published in 2012, 2014 and 2015 in high impact general medicine journals**

| **Search** | **Search terms** |
| --- | --- |
| #1 | Meta-analysis[Publication Type] |
| #2 | (New England Journal of Medicine[Journal]) OR (Lancet[Journal]) OR (JAMA[Journal]) OR (Ann Intern Med[Journal]) OR (BMJ[Journal]) OR (Arch Intern Med[Journal]) OR (PLOS Med[Journal]) OR (JAMA Intern Med[Journal]) OR (J Cachexia Sarcopenia Muscle [Journal]) OR (BMC Med[Journal]) OR (Mayo Clin Proc) |
| #3 | #1 AND #2 |
| #4 | #3 Limits: ("2012/01/01"[Date - Publication] : "2012/12/31"[Date - Publication]) OR ("2014/01/01"[Date - Publication] : "2014/12/31"[Date - Publication]) OR ("2015/01/01"[Date - Publication] : "2015/12/31"[Date - Publication]) |
